# Safety of 3 Tesla Magnetic Resonance Imaging in Patients with Sickle Cell Disease

**DOI:** 10.1155/2021/5531775

**Published:** 2021-05-11

**Authors:** Olivia Justice, Lori C. Jordan, Chelsea A. Lee, Niral J. Patel, Spencer Waddle, Sumit Pruthi, L. Taylor Davis, Adetola A. Kassim, Manus J. Donahue

**Affiliations:** ^1^Department of Radiology, Vanderbilt University Medical Center, Nashville, TN, USA; ^2^Department of Pediatrics, Division of Pediatric Neurology, Vanderbilt University Medical Center, Nashville, TN, USA; ^3^Department of Neurology, Vanderbilt University Medical Center, Nashville, TN, USA; ^4^Department of Pediatrics, Vanderbilt-Meharry Center for Excellence, Sickle Cell Disease, Vanderbilt University Medical Center, Nashville, TN, USA; ^5^Department of Internal Medicine, Division of Hematology/Oncology, Vanderbilt University Medical Center, Nashville, TN, USA; ^6^Department of Psychiatry, Vanderbilt University Medical Center, Nashville, TN, USA

## Abstract

Sickle cell disease (SCD) is a well-characterized hemoglobinopathy affecting more than 20 million individuals worldwide and carries an increased risk of cerebral vasculopathy, cerebral infarct, and stroke. As mechanisms of cerebral infarction in SCD are partly attributable to microvascular vaso-occlusive crises, manifesting as altered cerebral blood flow and associated impaired oxygen delivery, magnetic resonance imaging (MRI) methods that can quickly provide a comprehensive perspective on structural and functional disease status, without exogenous contrast administration or ionizing radiation, have emerged as crucial clinical tools for surveillance. However, early *ex vivo* MRI work in suspended erythrocytes containing hemoglobin S at 0.35 Tesla (T) suggested that sickled erythrocytes can orient preferentially in the presence of an external magnetic field, and as such, it was suggested that MRI exams in sickle cell hemoglobinopathy could induce vaso-occlusion. While this observation has generally not impacted clinical imaging in individuals with SCD, it has led to resistance for some sickle cell studies within the engineering community among some imaging scientists as this early observation has never been rigorously shown to be unconcerning. Here, we performed MRI at the clinical field strength of 3 T in 172 patients with SCD, which included standard anatomical and angiographic assessments together with gold standard diffusion-weighted imaging (DWI; spatial resolution = 1.8 × 1.8 × 4 mm; *b*-value = 1000 s/mm^2^) for acute infarct assessment (performed approximately 20 min after patient introduction to the field isocenter). The presence of vasculopathy, as well as chronic and acute infarcts, was evaluated by two independent board-certified radiologists using standard clinical criteria. In these patients (52.3% female; mean age = 19.6 years; age range = 6–44 years), hematocrit (mean = 25.8%; range = 15–36%), hemoglobin phenotype (87.8% HbSS variant), presence of silent infarct (44.2%), and overt chronic infarct (13.4%) were consistent with a typical SCD population; however, no participants exhibited evidence of acute infarction. These findings are consistent with 3 T MRI not inducing acute infarction or vaso-occlusion in individuals with SCD and suggest that earlier low-field *ex vivo* work of erythrocytes in suspension is not a sufficient cause to discourage MRI scans in patients with SCD.

## 1. Introduction

Sickle cell disease (SCD) is a genetically inherited blood disorder affecting approximately 300,000 new cases annually worldwide [1, 2]. SCD comprises a range of sickle phenotypes (HbSC, HbSS, HbS*β*^0^, HbS*β*^+^, HbSE, and HbSD) [3, 4], whereby the most common and severe is sickle cell anemia (SCA) characterized by the presence of hemoglobin (Hb)-S (HbSS). The presence of the HbSS mutation contributes to both vaso-occlusion as clustered, sickled erythrocytes restrict blood delivery through primarily microvasculature and also hemolysis, resulting in anemia and reduced oxygen delivery to tissues. These factors can manifest as pain crises, organ failure, and chronic anemia and neurologically as cerebral infarction and/or cognitive impairment [1, 5].

Magnetic resonance imaging (MRI), most commonly performed at a field strength of 1.5–3.0 Tesla (T), is often used in patients with SCD to evaluate prior infarct and more recently to evaluate mechanisms that subserve tissue oxygen [5]. MRI does not require exogenous contrast or ionizing radiation and therefore is well suited for surveillance imaging or therapy response evaluation. MRI is used frequently in both children and adults for infarct [5–7] and vasculopathy [8, 9] assessment, to evaluate iron loading and accumulation [10, 11], and studies have also reported evidence of acute infarcts visible on MRI in a small subset of children with SCD, which occurred with or without recent clinical symptoms [12, 13].

Some resistance, largely with imaging scientists, remains regarding the safety of MRI in patients with SCD despite widespread acceptance of MRI amongst clinicians and clinical researchers. This resistance is attributable to a study published in 1985 in *Investigative Radiology*, in which it was reported that erythrocytes drawn from individuals with SCA and scanned *ex vivo* in suspension at a field strength of 0.35 T align orthogonal to the external magnetic field, and as such, it is possible that this preferential orientation could contribute to an elevated risk of steno-occlusion if similar effects are present *in vivo* [14]. Despite more than three decades passing since this original work, no study has provided *in vivo* data on the potential safety of MRI in patients with SCD for not inducing acute infarcts. Importantly, large clinical trials that include neuroimaging in asymptomatic participants, such as the pediatric silent cerebral infarct transfusion (SIT) trial [13, 15], found that 10 out of 771 children with MRI had evidence of acute silent cerebral ischemia (confirmed by diffusion-weighted imaging, DWI) during scheduled, study-related MRIs not obtained for clinical indications and without stroke symptoms. These were termed acute silent cerebral ischemic events [13]. While 90% of these patients did not have a document report of medical illness two weeks prior to the MRI, some patients may have been in acute vaso-occlusive crises, and as such, it was not possible to discern whether these infarcts were due to the imaging procedure or had initiated before the procedure. A separate study also reported evidence of acute silent cerebral infarcts in seven children scanned with MRI [12]. The overarching assumption is that these observed acute infarcts are coincidental with the MRI as silent cerebral infarcts are common in individuals with sickle cell disease [15], and the imaging exam simply corresponded with the time of an ischemic event.

However, no prospective study has rigorously evaluated the presence of acute ischemic events in adults and children with SCD. To gain additional objective information on MRI safety in participants with SCD, here we apply DWI-MRI in stable pediatric and adult SCD participants to evaluate the primary study hypothesis that MRI performed at the highest clinical field strength of 3 T does not induce acute infarction *in vivo* in patients with SCD.

## 2. Methods

### 2.1. Demographics and Recruitment

All participants (*n* = 172) provided informed, written consent in accordance with the ethical standards and approval of the Vanderbilt University Medical Center Institutional Review Board. Participants comprised both adults (≥18 years of age) and children (6–17 years of age) with SCD (including phenotype HbSS, HbSC, or HbS*β*^0^) and were recruited from a comprehensive SCD clinic as part of a larger prospective study of hemometabolic imaging indicators of stroke risk. Participants were required not to have received medical attention for vaso-occlusive crises within the past seven days, and, if on chronic transfusion, to have been scanned at least three weeks since their last transfusion when their hematocrit was near nadir. Hematocrit and hemoglobin phenotype were evaluated via venipuncture within seven days of the MRI protocol.

### 2.2. Acquisition

DWI was applied to evaluate the primary study hypothesis, whereas standard anatomical T_2_-weighted fluid-attenuated inversion recovery (FLAIR) and T_1_-weighted MRI and time-of-flight magnetic resonance angiography (MRA) were applied in sequence to evaluate chronic infarct and vasculopathy extent, respectively. All imaging was performed using body coil radiofrequency transmission and phased-array SENSE reception at a field strength of 3 Tesla (Philips Healthcare, Best, Netherlands).

DWI was performed approximately 20 min after introducing the participant to the isocenter of the scanner and with a standard protocol with field view (FOV) = 230 × 230 × 129 mm, spatial resolution = 1.8 × 1.8 × 4 mm, and *b*-values of 0 and 1000 s/mm^2^. Acute lesions will appear on DWI minutes after a stroke occurs [16, 17]. In addition, FLAIR MRI was performed in the axial (FOV = 184 × 230 × 134 mm) and coronal (FOV = 161 × 199 × 200 mm) planes with a common spatial resolution of 0.9 × 1.1 × 3 mm for chronic infarct determination. Intracranial time-of-flight magnetic resonance angiography (spatial resolution = 0.6 × 0.6 × 1.4 mm^3^; 3D gradient echo; repetition time/echo time = 23/3.5 ms) and cervical time-of-flight magnetic resonance angiography (spatial resolution = 0.9 × 0.9 × 3.0 mm^3^; 2D gradient echo; repetition time/echo time = 18.6/3.2 ms) were performed for vasculopathy determination.

### 2.3. Analysis

Cervical and major intracranial vessels for each participant were assessed for vasculopathy by two board-certified radiologists as previously described [18]. Each vessel was graded as mild stenosis (0–50%), moderate stenosis (51–69%), severe stenosis (70–99%), or occlusion. Severity of intracranial vasculopathy was graded by the worst vessel seen as mild, moderate, severe, or occluded. Disagreements were resolved by consensus.

Infarcts were classified by the same radiologists. First, the scan was judged as normal or abnormal. If abnormal, the lesions were assigned as either (i) nonspecific white matter hyperintensity if <3 mm in diameter [19] or (ii) focal, discrete ischemic infarcts if ≥3 mm and visible in at least two planes of T_2_-weighted FLAIR MRI (axial and coronal). Infarcts were classified as silent using clinical radiological criteria defined above and if no neurological symptoms or signs corresponding to the location of infarcts were detectable following a neurological history or exam by a board-certified neurologist (LCJ; experience >15 years) [13, 15].

### 2.4. Statistical Considerations

The goal of this study was to report the prevalence of acute silent infarction in a cohort of SCD participants without acute vaso-occlusive crises. Descriptive statistics including mean and ranges for continuous variables (i.e., age and hematocrit) were calculated, along with percent for categorical variables (i.e., sex, acute infarct on DWI MRI, chronic infarct on FLAIR MRI, vasculopathy extent, children or adults, and hemoglobin phenotype). Results are presented both in a stratified manner for biological sex and in a cumulative (i.e., all volunteers) manner. An unpaired Student's *t*-test and chi-squared test were used for evaluating differences in continuous and categorical variables, respectively, between groups. In all cases, two-sided *p* < 0.05 was required for reporting significance.

Finally, because prior infarcts and cerebral vasculopathy are risk factors for new infarcts [6, 18], to provide a description of how the prevalence of these risk factors changed with age, we plotted the cumulative presence of participants with silent cerebral infarct, acute infarct, chronic overt stroke, and vasculopathy with age. This subanalysis provided information on the approximate prevalence of these risk factors across the SCD lifespan.

## 3. Results

The enrolled cohort consisted of 172 SCD participants, comprised primarily sickle cell anemia (88% HbSS), with a smaller subset that comprised HbS *β*^0^thalassemia (10% HbS*β*^0^) and milder sickling (2% HbSC). Of these, 52.9% of participants were adults, and 47.1% were children. The mean age of participants in the study was 19.6 years (age range = 6–44 years). 97% of participants were Black, and 3% were Middle Eastern or Asian Indian. Summary statistics for all measures are provided in Table 1.

As required by the inclusion criteria, no participants reported a vaso-occlusive crisis within the past seven days, and none were hospitalized for related indications, including blood transfusion, within seven days before the scan. As such, all participants met criteria for being stable without known, recent acute events.

Of these participants, the mean hematocrit was 25.8% (range = 15%–36%). Hematocrit was mild but significantly reduced in female vs. male patients (*p*=0.002). This breakdown is consistent with typical ranges and hemoglobin phenotypes present in most North American sickle cell disease clinics. Of these participants, 9.3% met criteria for vasculopathy on MRA. On T_1_-weighted and T_2_-weighted FLAIR MRI, 76 participants had evidence of SCIs, and 23 participants had evidence of chronic infarcts from overt stroke. The number of chronic silent infarcts was statistically higher in the male vs. female participants (chi-square test; *p*=0.018).

**Figure 1:**
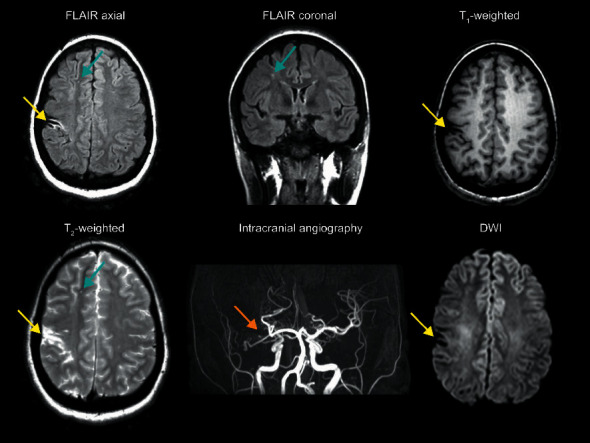
Representative scans from the multimodal magnetic resonance imaging protocol from a Black 9-year-old female with hemoglobin SS phenotype. Chronic stroke is apparent (yellow arrows), with additional right parietal and frontal lobe chronic silent cerebral infarcts (blue arrows), which largely colocalize with patchy T_2_-hyperintense foci. On intracranial angiography, there is near-complete occlusion of the terminus of the right internal carotid artery with a very short proximal portion of the right M1 segment visible (orange arrow). Extensive collateral vessels are identified extending into the basal ganglia region on the right side. No evidence of acute infarct is visible on diffusion-weighted imaging (DWI).

No participants met criteria for radiological indicators of acute stroke on DWI.  Figure 1 displays the quality of imaging data used in the study, summarized from a representative participant with chronic silent infarcts but without evidence of acute infarct. These data are consistent with MRI, performed at the highest clinical field strength of 3 Tesla, not inducing acute infarction in individuals with SCD.

**Figure 2:**
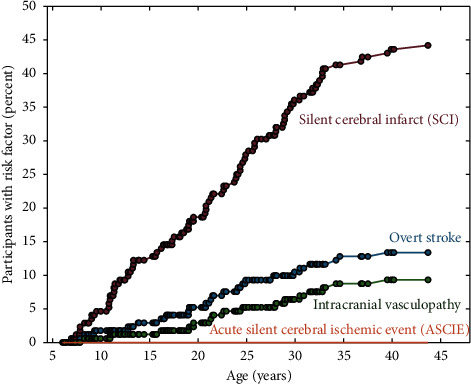
Cumulative incidence of radiological risk factors with age over the 172 participants. Note that no participants exhibited evidence of acute silent cerebral ischemic events on diffusion-weighted imaging (DWI).

Figure 2 summarizes the cumulative incidence of an event (SCI, overt stroke, observed acute infarct, or vasculopathy) with age. While SCI, overt stroke, and vasculopathy were all observed to increase with increasing age, with the slope being highest for SCIs, there was no evidence of an increase in acute infarct prevalence with increasing age.

## 4. Discussion

Despite the relative routine use of MRI in patients with SCD, prior large studies have observed limited evidence of DWI lesions [13], and one earlier *ex vivo* evidence suggests that sickle red blood cells' orientation in the presence of an external magnetic field may lead to vaso-occlusive crises [14] However, these prior studies have not controlled for whether patients were in or had recent vaso-occlusive crises that may have led to the DWI being positive for acute infarct, and as such, it was not possible to definitively determine if it was the MRI or the recent clinical history and underlying disease process that accounted for the acute infarct. Given the low prevalence of these acute infarcts, it has been commonly assumed that the MRI itself was not the cause of these events; however, this has not been definitively shown, and prior work suggesting this possibility has not been directly addressed [14]. Here, we performed 3 Tesla MRI in 172 SCD patients in stable conditions without known vaso-occlusive crises. The findings are that in no patients were acute lesions detectable on MRI. These findings provide support for the safety of MRI, performed at the highest available clinical strength of 3 Tesla, in patients with sickle cell disease.

These findings should be considered in light of the original study from 1985 which suggested that MRI may be unsafe in patients with SCD [14]. In this original study, *in vivo* measurements were not performed, and rather, it was only determined *ex vivo* that sickled erythrocytes in suspension have a preferential orientation at a low magnetic field strength of 0.35 T. While it is logical that this orientation should also exist at higher magnetic field strengths, the current data suggest that this biophysical phenomenon does not translate *in vivo* to vaso-occlusion and infarction. The likely reason for this is that the flow characteristics *in vivo* and the associated vascular orientation determine the erythrocyte orientation much more than the static magnetic field, and as such, infarction secondary to the magnetic field is not observed.

The study should also be considered in light of several limitations. First, the study included only 172 total participants. However, thousands of clinical MRIs are performed annually in individuals with SCD without incident as the American Society of Hematology guidelines recommend screening MRI to assess for silent cerebral infarcts in SCD at least once in childhood and adulthood [20]. Second, we did not find any difference between participants with different sickle cell phenotypes. However, as sickle phenotypes beyond HbSS are relatively rare, the sample size for these other phenotypes is largely insufficient to make claims regarding specific phenotypes. Finally, DWI was performed approximately 20 min after introducing the patient to the isocenter of the scanner. While infarction on DWI should be apparent within several minutes, we cannot rule out that longer waiting times may have yielded different results if ischemia was more mild or collateral flow more robust. However, it should be noted that typical noncontrast head MRI protocols, as are performed in SCD patients, have a duration of approximately 20–25 minutes, and therefore, this time is roughly indicative of a clinical exam.

In conclusion, we performed 3 Tesla MRI in 172 stable SCD participants with no recent history of vaso-occlusive crisis and representative of a typical cohort of pediatric and adult patients; findings are consistent with 3 T MRI not inducing acute infarction or vaso-occlusion in individuals with SCD and suggest that earlier low-field *ex vivo* work should not discourage MRI scans from being performed in either children or adults with SCD.

## Figures and Tables

**Table 1:** Sickle cell disease (SCD) participant demographics and percentage with acute, overt, and silent stroke.

	Female	Male	Combined
Mean age (range) (years)	19 (6–44)	20 (6–37)	19.6 (6–44)
Total number of participants	90 (52.3)	82 (47.7)	172
Number of adults ≥18 years (percent)	43 (47.8)	48 (58.5)	91 (52.9)
Number of children <18 years (percent)	47 (52.2)	34 (41.5)	81 (47.1)
Total hematocrit (range) (percent)	26.1 (15–36)	26.7 (17–35)	25.8 (15–36)
Number with acute infarct present (percent)	0	0	0
Number with chronic silent infarct present (percent)	32 (35.6)	44 (53.7)	76 (44.2)
Number with chronic overt stroke present (percent)	14 (15.6)	9 (11.0)	23 (13.4)
Number with vasculopathy present (percent)	9 (10.0)	7 (8.5)	16 (8.5)
Number with HbSS phenotype (percent)	77 (85.6)	74 (90.2)	151 (87.8)
Number with HbS*β*^0^ phenotype (percent)	10 (11.1)	7 (8.5)	17 (9.9)
Number with HbSC phenotype (percent)	3 (3.3)	1 (1.2)	4 (2.3)

## Data Availability

The data used to support the findings of this study will be made available to researchers from the corresponding author upon request, with up-to-date HIPAA training.
